# Direct electrical measurement of tube‐voltage waveforms in mammography systems using ALDEN‐type high‐voltage connectors

**DOI:** 10.1002/mp.70477

**Published:** 2026-05-14

**Authors:** Toru Negishi, Kiyomitsu Shinsho, Shinji Abe, Izumi Ogura

**Affiliations:** ^1^ Graduate School of Human Health Sciences Tokyo Metropolitan University Arakawa‐ku Tokyo Japan

**Keywords:** ALDEN connector, direct waveform measurement, mammography, tube voltage waveform

## Abstract

**Background:**

Accurate tube‐voltage characterization is essential for mammography quality assurance and estimation of mean glandular dose (MGD). In mammography systems equipped with ALDEN‐type high‐voltage connectors, direct electrical access to the tube‐voltage circuit has historically been restricted, limiting evaluation primarily to non‐invasive measurement methods.

**Purpose:**

To develop and evaluate a direct‐connection adapter enabling invasive acquisition of tube‐voltage waveforms in mammography systems using ALDEN‐type high‐voltage connectors and to compare waveform characteristics with those obtained using a non‐invasive multimeter.

**Methods:**

A direct‐connection adapter‐based measurement system was developed to provide electrical access to the tube‐voltage circuit without modifying the primary conduction path. Tube‐voltage measurements were performed using a commercial tube‐voltage/tube‐current meter incorporating a fixed high‐voltage divider (1:20 000). Tube‐voltage waveforms were measured in three clinical mammography systems under multiple target/filter configurations using both the invasive method and a commercially available non‐invasive multimeter (RaySafe X2). Tube‐voltage ripple was quantified from time‐resolved voltage data obtained under identical exposure conditions.

**Results:**

Mean tube‐voltage values obtained using invasive and non‐invasive approaches showed approximately linear agreement with preset voltage. However, ripple magnitudes differed substantially under certain beam conditions. The invasive method yielded ripple values ranging from 2.5% to 6.2%, whereas non‐invasive measurements reached up to 48.5% under specific configurations.

**Conclusions:**

Direct electrical measurement of tube‐voltage waveforms in mammography systems using ALDEN‐type high‐voltage connectors was demonstrated using the proposed adapter. Because the invasive method directly measures the electrical high‐voltage waveform without signal reconstruction, it provides an electrical reference for evaluating potential variability in non‐invasive waveform measurements and may support generator characterization and waveform‐based investigations in research and equipment evaluation settings.

## 1 INTRODUCTION

Accurate measurement of tube voltage is essential for maintaining image quality and estimating mean glandular dose (MGD) in mammographic X‐ray systems.^1^, ^2^, ^3^, ^4^, ^5^, ^6^ National and international guidelines, including the Mammography Guideline, the Mammography Quality Control Manual, and IEC 61223‐3‐2, consistently emphasize the importance of tube‐voltage accuracy and waveform assessment as fundamental components of quality assurance.^1^, ^2^, ^3^, ^4^, ^5^, ^6^, ^7^, ^11^ Tube voltage strongly influences beam quality, half‐value layer (HVL), and dose estimation, and even small deviations in tube voltage may influence MGD estimation, as described in IEC 61223‐3‐2.^11^

In clinical practice, tube voltage is typically evaluated using non‐invasive X‐ray meters.^8^, ^9^ These devices provide convenient and reproducible measurements and are widely used for routine quality control. However, non‐invasive systems generally derive tube‐voltage information indirectly from detected radiation signals, and their temporal resolution and waveform reconstruction methods may influence the representation of high‐frequency characteristics. Detailed waveform components—such as voltage ripple, transient overshoot, rise behavior, and automatic exposure control (AEC)—related modulation—are therefore challenging to evaluate with direct electrical access to the high‐voltage circuit.

Direct tube‐voltage measurement in mammographic systems has historically been technically challenging because many systems employ ALDEN‐type high‐voltage connectors that prevent insertion of measurement probes into the tube circuit. Although generator‐specific ripple characteristics have been reported previously,^12^ direct waveform acquisition in mammography systems using ALDEN‐type high‐voltage connectors has not been demonstrated. This limitation has represented a longstanding obstacle in comprehensive generator characterization and waveform‐based quality assessment.

To address this technical barrier, we developed a dedicated direct‐connection adapter designed for ALDEN‐type connectors, enabling invasive yet non‐modifying access to the high‐voltage circuit. This Technical Note describes the design concept and implementation of the adapter and presents fundamental performance evaluations, including direct waveform acquisition in clinical inverter‐type mammography systems. The purpose of this work is to demonstrate the feasibility of direct waveform measurement in mammography systems using ALDEN‐type high‐voltage connectors and to evaluate its potential contribution to waveform‐based generator assessment. From a measurement science perspective, direct electrical access provides a reference framework for evaluating potential system‐dependent variability in non‐invasive waveform measurements.

## 2 METHODS

### 2.1 Adapter design for direct high‐voltage connection

A direct‐connection adapter‐based measurement system was developed to enable access to the tube‐voltage circuit in mammographic X‐ray systems equipped with ALDEN‐type high‐voltage connectors (Alden Products, Inc., USA). The adapter incorporates an ALDEN‐502 receptacle on the generator input side and an ALDEN P503BH “L” tripolar plug on the tube side, allowing insertion between the generator and tube without modifying the clinical system. Internal shielding and high‐voltage insulation materials were incorporated to minimize leakage current and waveform distortion.

Tube‐voltage measurements were performed using a commercial tube‐voltage/tube‐current meter (AB‐2015E, Toreck Co., Ltd., Japan), which incorporates a fixed high‐voltage divider (1:20 000) and a current‐sensing resistor. The developed adapter functions as an interface that enables electrical access to the high‐voltage circuit without modifying the primary conduction path. The selected division ratio is consistent with commercially available X‐ray tube voltage and current meters used for quality assurance, such as the AB‐2015E (Toreck Co., Ltd., Japan), and with calibration methodologies based on calibrated high‐voltage dividers described in JIS Z 4511 and ISO 4037‐4.

**FIGURE 1:**
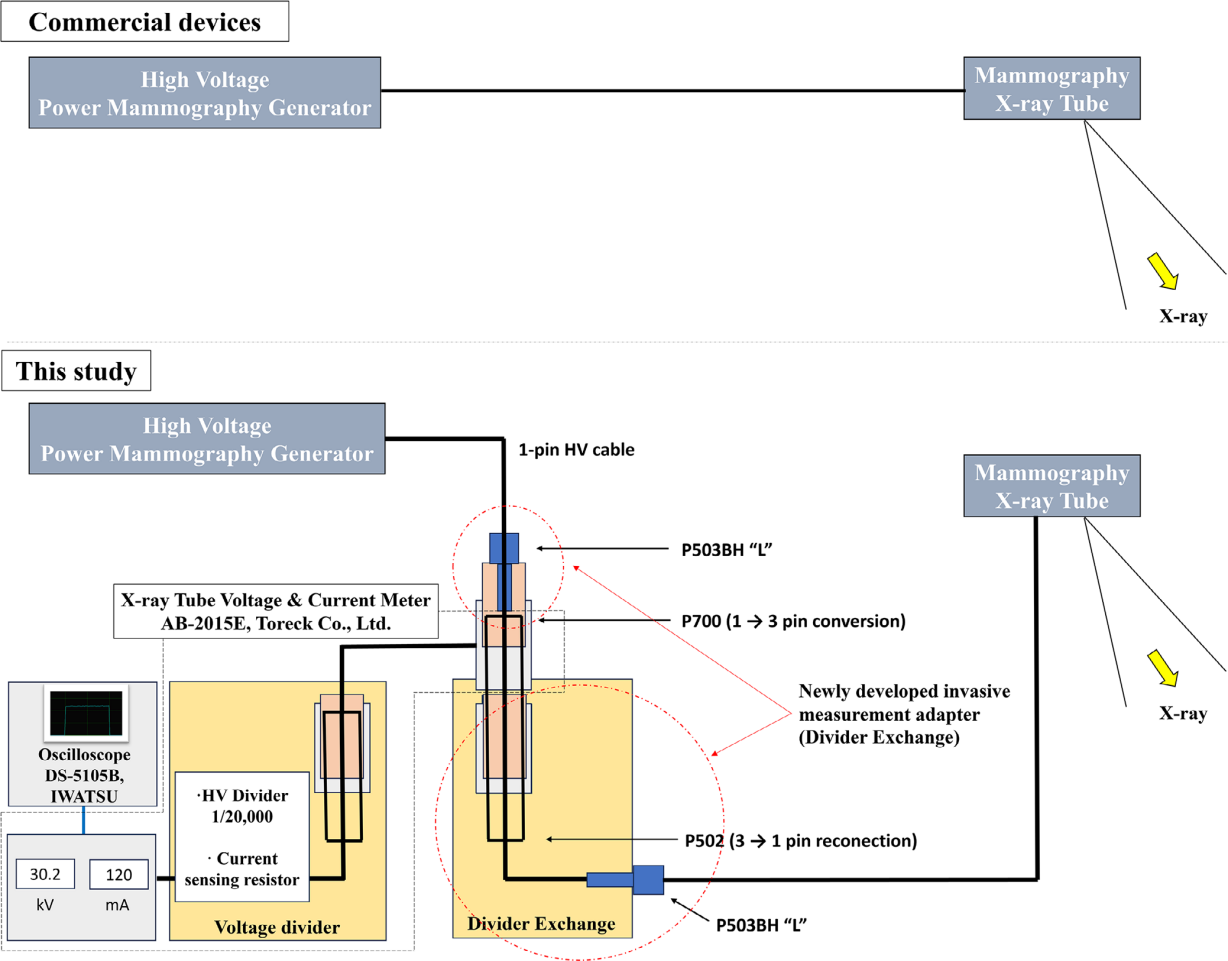
Schematic configuration of the invasive measurement system developed in this study.

The adapter was designed to preserve waveform fidelity up to 50 kHz to accommodate inverter‐type mammography generators. Figure 1 illustrates the complete measurement configuration and internal functional components of the developed invasive measurement system.

Because the mammography system employs a single‐pin ALDEN‐type high‐voltage configuration, direct connection to a tripolar measurement system is not possible. Therefore, a 1‐to‐3 pin conversion interface was incorporated to enable compatibility with the measurement unit. After measurement, the three conductors were reconfigured to restore the original single‐pin system configuration.

Importantly, the main high‐voltage conduction path remains unchanged during measurement, while a parallel low‐voltage branch is used for waveform acquisition. This configuration enables safe signal branching without altering the clinical generator circuitry.

Installation and removal of the adapter were performed by qualified service engineers in accordance with manufacturer procedures. This approach is intended for equipment evaluation and research purposes rather than routine clinical quality control.

Low‐voltage waveform outputs from the commercial measurement device (AB‐2015E) were recorded using a digital oscilloscope (DS‐5105B, Iwatsu, Japan) with a bandwidth of 50 MHz and a maximum sampling rate of 1 GS/s (single‐channel mode). During waveform acquisition, a sampling rate of 500 MS/s or higher was used. These specifications exceed the evaluated frequency range (≤50 kHz) by more than three orders of magnitude, ensuring sufficient temporal resolution and minimizing measurement‐induced distortion. The oscilloscope input scale corresponds to the divided low‐voltage signal, and the actual tube voltage was calculated by applying the fixed divider ratio (1:20 000). Therefore, the oscilloscope performance was not a limiting factor in ripple quantification.

The system was inserted between the mammography generator and X‐ray tube equipped with ALDEN‐type high‐voltage connectors. A 1‐to‐3 pin conversion interface enables compatibility with the tripolar measurement unit, and the original single‐pin configuration is restored after measurement.

Tube‐voltage measurements were performed using a commercial tube‐voltage/tube‐current meter (AB‐2015E), which incorporates a fixed high‐voltage divider (1:20 000) and a current‐sensing resistor. The developed adapter provides electrical access to the high‐voltage circuit and does not include voltage division components.

### 2.2 Evaluation using mammographic X‐ray systems

Performance was assessed using three clinical mammography systems manufactured by Canon Medical Systems:

1. Peruru (MGU‐1000D)
2. MGU‐100B
3. LaPlus (MGU‐1000D/NJ)

Measurements were performed under multiple target/filter combinations to assess waveform characteristics under different beam qualities. For Peruru (MGU‐1000D), Mo/Mo configurations were evaluated. For MGU‐100B, Mo/Rh configurations were examined. For LaPlus (MGU‐1000D/NJ), W/Rh and W/Ag configurations were examined.

The developed adapter was inserted into the anode‐side high‐voltage cable between the generator and the X‐ray tube, as illustrated in Figure 1. Tube‐voltage waveforms were directly measured using a tube‐voltage/tube‐current meter (AB‐2015D M2, Toreck Co., Ltd., Japan) and a digital oscilloscope (DS‐5105B, Iwatsu, Japan). Waveform height, exposure time, and tube‐voltage ripple percentage were determined using an A/D converter (NR‐2000, Keyence, Japan).

For each target/filter configuration, measurements were obtained under identical exposure parameters using both the proposed invasive measurement system and a commercially available non‐invasive X‐ray multimeter (RaySafe X2, RaySafe AB, Sweden). This enabled comparative evaluation of waveform representation and ripple characteristics under identical exposure conditions.

In waveform measurements, the oscilloscope voltage scale represents the divided low‐voltage signal obtained from the built‐in high‐voltage divider. The actual tube voltage was calculated by multiplying the measured voltage by the fixed divider ratio (1:20 000). Scale indicators shown on the oscilloscope display correspond to the input range of each channel and do not represent the actual tube voltage.

Tube‐voltage ripple (%) was calculated as

$$\mathrm{ripple}\left(\%\right)=\frac{V\max-V\min}{V\mathrm{mean}}\times 100$$

where $V_{\max}$ and $V_{\min}$ represent the maximum and minimum tube‐voltage values during the steady‐state portion of the exposure, excluding rise and fall intervals, and $V_{\mathrm{mean}}$ represents the mean tube voltage over the same interval.

Among the evaluated beam qualities, the W/Rh configuration of the LaPlus system was selected for detailed waveform comparison because it is a commonly used mammography setting and exhibited measurable differences in ripple characteristics between measurement approaches.

### 2.3 Evaluation of non‐invasive measurement dependence on system configuration

To investigate the dependence of non‐invasive waveform measurements on generator type and beam quality, tube‐voltage ripple values obtained using the non‐invasive multimeter (RaySafe X2) were compared across different mammography systems and target/filter combinations.

Measurements were performed under identical exposure parameters for each configuration. The resulting ripple values were compared with those obtained using the invasive measurement system to evaluate consistency and system‐dependent variation.

## 3 RESULTS

### 3.1 Direct versus non‐invasive waveform comparison

**FIGURE 2:**
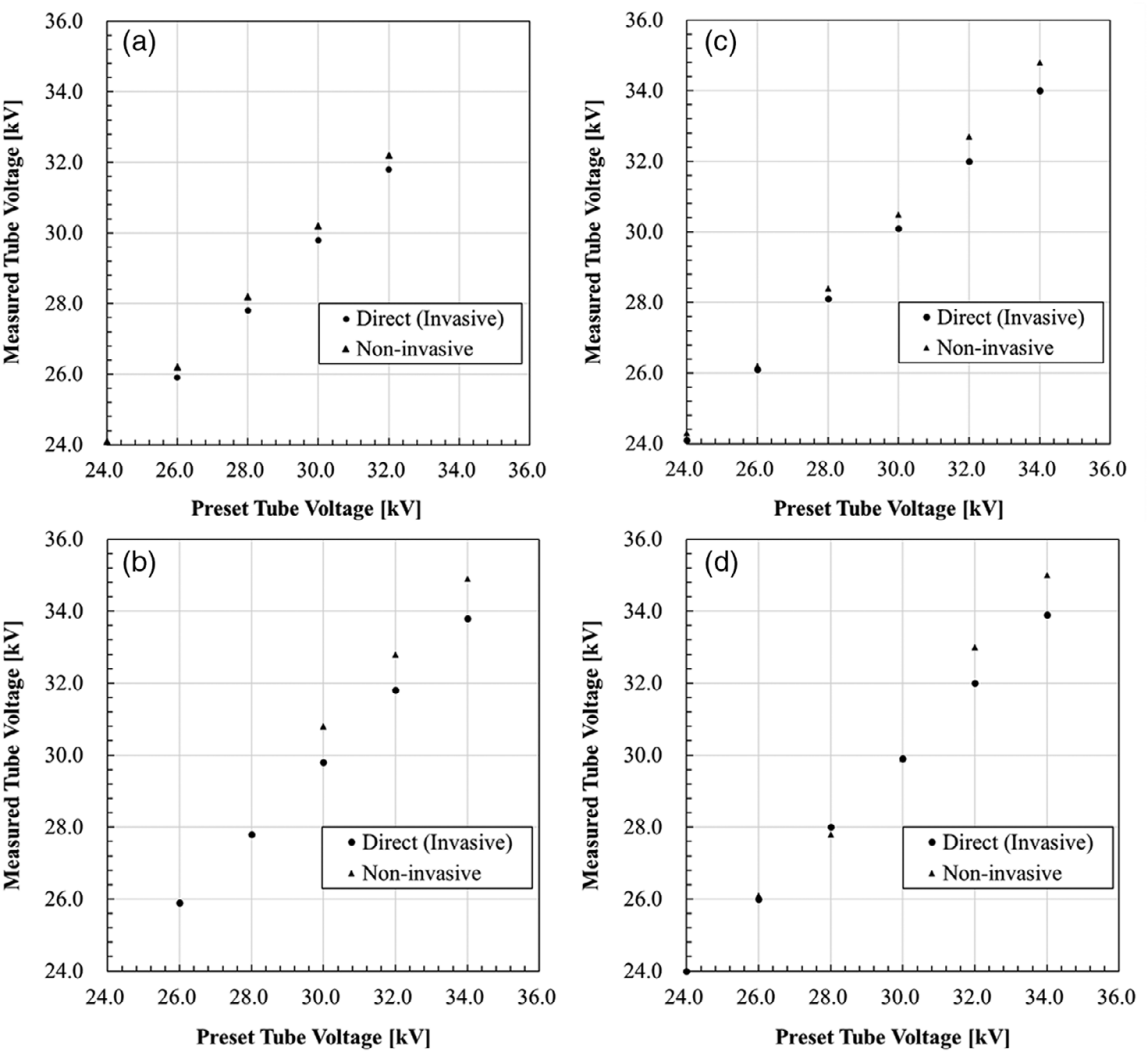
Comparison of tube‐voltage measurements obtained using invasive (direct) and non‐invasive methods as a function of preset tube voltage. Data are shown for four target/filter configurations: (a) Peruru system, Mo/Mo; (b) MGU‐100B system, Mo/Rh; (c) LaPlus system, W/Rh; and (d) LaPlus system, W/Ag. The invasive measurements were performed using the developed direct‐connection adapter with a fixed high‐voltage divider (1:20 000), whereas the non‐invasive measurements were obtained using a commercial multimeter (RaySafe X2). For each configuration, identical exposure conditions were used for both measurement approaches. The invasive and non‐invasive measurements show approximately linear relationships with preset tube voltage, indicating generally consistent trends in mean tube‐voltage estimation. However, subsequent waveform analysis revealed differences in ripple magnitude and temporal waveform characteristics between the two measurement methods.

Representative tube‐voltage waveforms obtained using the invasive (direct) method and the non‐invasive multimeter (RaySafe X2) are shown in Figure 2 for four target/filter configurations: Peruru (Mo/Mo), MGU‐100B (Mo/Rh), LaPlus (W/Rh), and LaPlus (W/Ag).

Across all configurations, both measurement approaches demonstrated approximately linear relationships between preset and measured tube voltage (Figure 2). The mean tube‐voltage values obtained using the two methods were generally consistent in trend. However, differences were observed in the time‐resolved waveform characteristics and in the ripple magnitudes derived from the two approaches. The mean tube‐voltage differences between invasive and non‐invasive measurements were within approximately 1 kV across all evaluated conditions, indicating that overall kV accuracy was largely preserved despite differences in ripple representation.

The ripple percentages corresponding to the same exposure conditions are summarized in Table 1. Missing values correspond to exposure conditions outside the specified operating range of the RaySafe X2 MAM sensor. The invasive measurement method yielded ripple values ranging from 2.5% to 6.2% across all generator systems and beam configurations. In contrast, ripple values derived from the non‐invasive multimeter were consistently higher under most conditions, reaching up to 48.5% for the Mo/Rh configuration of the MGU‐100B system. For W/Rh and W/Ag beam conditions, ripple values obtained using the non‐invasive system were typically several times greater than those derived from direct electrical waveform acquisition. In certain configurations, ripple values could not be obtained using the non‐invasive system because the exposure conditions exceeded its specified operating range.

**TABLE 1:** Ripple percentages (%) derived from time‐resolved tube‐voltage waveforms under identical exposure conditions corresponding to Figure 2. Values are shown for the invasive (direct) measurement method and the non‐invasive multimeter (RaySafe X2) across different generator systems and target/filter configurations. Ripple was calculated from the measured voltage waveforms as described in Section 2.2. Missing values in the non‐invasive measurements indicate operating conditions outside the specified measurement range of the multimeter.

System/target–filter	Preset tube voltage [kV]	Invasive (this study) [%]	Non‐invasive (RaySafe X2) [%]
(A) Peruru, Mo/Mo	26	4.62	11.19
	28	4.29	10.28
	30	4.00	12.71
	32	3.70	15.83
	34	—	—
(B) MGU‐100B, Mo/Rh	26	—	—
	28	—	—
	30	4.00	48.45
	32	2.50	36.17
	34	3.49	33.65
(C) LaPlus, W/Rh	26	6.15	21.31
	28	4.29	19.69
	30	5.26	15.11
	32	3.70	15.78
	34	3.53	18.30
(D) LaPlus, W/Ag	26	6.15	10.95
	28	4.29	29.37
	30	5.26	27.78
	32	3.70	20.85
	34	3.53	15.80

These findings indicate that ripple values derived from non‐invasive systems may depend on generator characteristics, beam quality, and the signal processing or reconstruction algorithms employed by the device. Because the invasive method directly acquires the high‐voltage waveform without signal reconstruction, it provides an electrical reference for evaluating potential system‐dependent variability in non‐invasive ripple representation.

### 3.2 Detailed waveform comparison under W/Rh beam conditions (LaPlus system)

**FIGURE 3:**
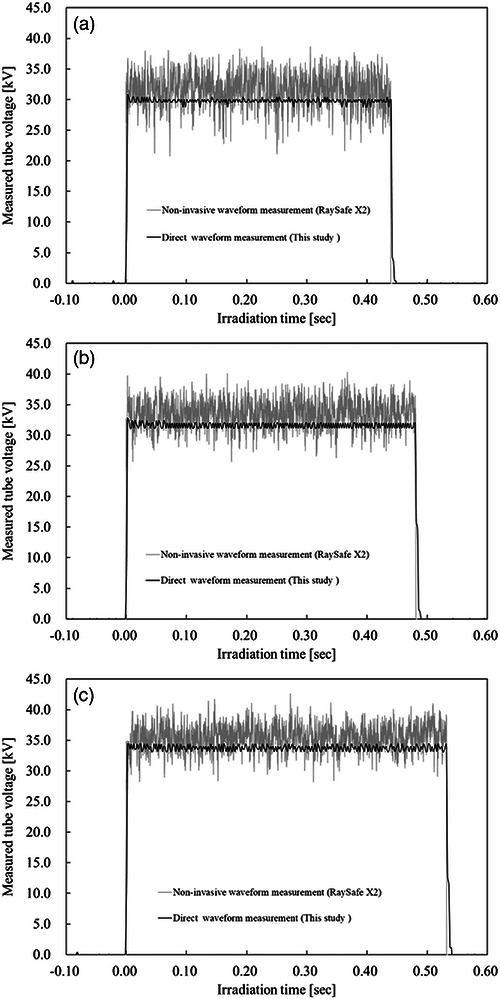
Time‐resolved tube‐voltage waveforms measured under W/Rh beam conditions in the LaPlus mammography system. Waveforms are shown for preset tube voltages of (A) 30 kV, (B) 32 kV, and (C) 34 kV. The black traces represent invasive (direct) measurements obtained using the fixed high‐voltage divider (1:20 000), whereas the gray traces represent non‐invasive measurements acquired using a multimeter (RaySafe X2). Identical time and voltage scales were applied to enable direct comparison between the two measurement approaches. The invasive measurements resolve periodic ripple structures corresponding to generator switching cycles, whereas the non‐invasive measurements show larger apparent ripple amplitudes and less clearly defined waveform structures.

Figure 3 presents time‐resolved tube‐voltage waveforms for the LaPlus system under the W/Rh configuration at preset voltages of 30, 32, and 34 kV.

Across all voltage settings, the invasive measurement method resolved a stable and periodic ripple structure with consistent amplitude and temporal characteristics. The waveform exhibited regular oscillatory behavior corresponding to the generator switching cycle. The non‐invasive multimeter (RaySafe X2) also demonstrated periodic voltage variations; however, differences in ripple magnitude and waveform shape were observed relative to the directly measured electrical waveform. Differences in ripple magnitude were observed across all evaluated voltage settings, with particularly large discrepancies noted under certain exposure conditions. In addition, minor differences were observed in the representation of rise and fall transitions between the two measurement approaches.

These results indicate that time‐resolved voltage representation may vary depending on the measurement principle employed. Because the invasive method directly acquires the electrical high‐voltage waveform without signal reconstruction, it provides a reference for evaluating waveform characteristics under specific beam conditions. Although the average tube‐voltage levels showed generally consistent trends between the two measurement approaches, differences in ripple magnitude and temporal waveform structure were consistently observed under W/Rh beam conditions.

## 4 DISCUSSION

This study demonstrated the feasibility of direct electrical acquisition of tube‐voltage waveforms in mammographic systems equipped with ALDEN‐type high‐voltage connectors. Historically, such systems have limited access to the high‐voltage circuit, restricting evaluation to non‐invasive measurement techniques. The developed adapter enabled invasive yet non‐modifying access to the tube‐voltage signal, preserving the primary high‐voltage conduction path while providing a parallel low‐voltage output for waveform acquisition.

The measurement system was designed to support waveform acquisition in inverter‐type mammography generators, whose dominant switching frequency components are typically below several tens of kilohertz. The oscilloscope bandwidth and sampling rate used in this study were sufficient to resolve these waveform characteristics. Combined with the 50 MHz oscilloscope bandwidth and high sampling rate, the measurement system provided sufficient temporal resolution for reliable ripple quantification.

Although mean tube‐voltage values measured using the invasive and non‐invasive approaches showed generally consistent linear trends with preset voltage, the observed differences in ripple magnitude suggest that conventional kV accuracy evaluation alone may not fully capture waveform‐dependent variability in generator performance.

It is important to note that non‐invasive mammography meters estimate tube voltage indirectly from detected radiation signals, and their performance depends on beam quality, filtration, detector response, and internal signal‐processing algorithms. According to manufacturer documentation, supported target/filter combinations and measurement parameters are defined for specific configurations. Therefore, discrepancies in ripple magnitude should be interpreted in the context of differences in measurement principles rather than as direct evidence of generator instability. The high‐voltage divider used in this study operates as a passive linear element designed with distributed resistive and capacitive components to maintain frequency response characteristics. It scales the signal amplitude without selectively suppressing specific frequency components within the relevant frequency range. Therefore, the relative ripple characteristics are preserved, and the observed differences in ripple magnitude are unlikely to be caused by the divider itself.

Because the invasive method directly acquires the electrical high‐voltage waveform without signal reconstruction, it provides a reference framework for evaluating potential system‐dependent variability in non‐invasive waveform measurements. Although invasive and non‐invasive waveforms were compared in this study, the primary objective was not to evaluate measurement accuracy between the two methods. Non‐invasive systems estimate tube voltage indirectly from detected radiation signals, whereas the present adapter directly measures the electrical waveform within the high‐voltage circuit. Therefore, differences observed between the two approaches should be interpreted in the context of their fundamentally different measurement principles rather than as evidence of generator instability.

In mammography, tube‐voltage characteristics influence beam quality and MGD estimation. Although the present study did not directly evaluate dosimetric impact, MGD calculations are sensitive to beam quality parameters, including effective photon energy and half‐value layer (HVL). Consequently, waveform‐dependent variability in ripple representation may introduce secondary effects on dose estimation, even when mean tube‐voltage values appear consistent. However, the present study did not directly evaluate HVL or dosimetric impact, and further investigation is required to clarify the potential magnitude of such effects.

The present study has several limitations. First, measurements were performed on a limited number of generator models from a single manufacturer. Second, the non‐invasive comparison was performed using a single commercially available multimeter. Future investigations, including additional systems and measurement devices, would further clarify the generalizability of these findings.

## 5 CONCLUSION

This Technical Note demonstrated the feasibility of direct electrical acquisition of tube‐voltage waveforms in mammographic systems equipped with ALDEN‐type high‐voltage connectors using a custom‐designed adapter.

The developed system enabled time‐resolved measurement of tube‐voltage and ripple characteristics without modifying the primary high‐voltage conduction path. While mean tube‐voltage values obtained using invasive and non‐invasive methods showed generally consistent linear trends with preset voltage, differences were observed in derived ripple magnitudes and waveform representation under certain beam conditions.

Because the invasive approach directly measures the electrical high‐voltage waveform without signal reconstruction, it provides a reference framework for evaluating potential variability in non‐invasive waveform measurements. The proposed method may serve as a useful tool for generator characterization and waveform‐dependent investigations in research and equipment evaluation settings.

## CONFLICT OF INTEREST STATEMENT

The authors declare no conflicts of interest. No external company had any involvement in the study design, data acquisition, analysis, interpretation, or the decision to submit this manuscript.

## Data Availability

The data supporting the findings of this study are available from the corresponding author upon reasonable request.
